# Comparative Evaluation of Sodium Hypochlorite Gel Penetration Using Er,Cr:YSGG Laser and Passive Ultrasonic Activation After Apicoectomy: An In Vitro Study with Confocal Laser Scanning Microscopy

**DOI:** 10.3390/jcm13237050

**Published:** 2024-11-22

**Authors:** Joseph Di Franco, Haitham Elafifi Ebeid, Pablo Betancourt, Antonio Pallarés-Sabater, Alberto Casino Alegre

**Affiliations:** 1 Department of Endodontics and Restorative Dentistry, School of Medicine and Dentistry, Catholic University of Valencia, 46001 Valencia, Spain; joe.2008@icloud.com (J.D.F.); antonio.pallares@ucv.es (A.P.-S.); 2 Oral Surgery Department, University of Barcelona, 08907 Barcelona, Spain; 3 Department of Surgery, Faculty of Health and Sports Sciences (Dentistry), University of Zaragoza, 22006 Zaragoza, Spain; 4 Endodontic Laboratory, Center for Research in Dental Sciences (CICO), Faculty of Dentistry, Universidad de La Frontera, Temuco 4780000, Chile; pablo.betancourt@ufrontera.cl; 5 Department of Integral Adultos, Faculty of Dentistry, Universidad de La Frontera, Temuco 4780000, Chile; 6 Doctoral School, Catholic University of Valencia, 46001 Valencia, Spain

**Keywords:** laser-activated irrigation, Er,Cr:YSGG, passive ultrasonic activation, sodium hypochlorite, apicectomy

## Abstract

**Background:** Lasers from the erbium family have been investigated to activate irrigation with sodium hypochlorite (NaOCl), improving the disinfection depth of the dentinal tubules of the root canal walls during root canal treatment. However, the possibility of laser-activated irrigation (LAI) in retro-cavity preparation has not been investigated to the date. The aim of our experimental study is to evaluate the efficacy of NaOCl gel penetration inside the dentinal tubules when activated during retro-cavity preparation, comparing passive ultrasonic activation (PUI) and Er,Cr:YSGG LAI. Materials and **Methods:** Fifty extracted mature single-root human teeth were divided into four groups (control, PUI, and two LAI groups with different NaOCl concentrations). After conventional endodontic treatment and root end resection, NaOCl gel (impregnated with rhodamine dye for confocal laser scanning microscopy (CLSM) analysis) was applied and activated according to the study group. The penetration index and mean penetration length were measured using computer software. **Results:** Both penetration index and mean penetration length were found to have increased in the PUI group compared to the control samples. However, LAI had a better penetration that was statistically significant compared to both the PUI and control groups. The difference in NaOCl concentration in the laser groups did not affect the penetration values. **Conclusions:** Within the limitations of our in vitro study using NaOCl gel activation in the retro-cavity after apicectomy, Er,Cr:YSGG LAI significantly enhanced NaOCl gel penetration capacity compared to PUI, regardless of its concentration. LAI can enhance its penetration in a safe way, avoiding its extrusion to the surrounding periapical tissues.

## 1. Introduction

Endodontic apical microsurgery is a reliable option to address persistent periapical pathology in cases where non-surgical treatment is unsuccessful or not feasible [1]. This procedure offers a success rate of 80–90%, making it a viable alternative to non-surgical treatment [2]. However, residual filling materials and infected dentin can potentially remain in root canals and dentinal tubules, especially if the teeth have been previously instrumented and sealed [3].

The apical third of the root canal system is often the site of endodontic treatment failure [4]. Factors such as lateral canals, apical deltas, anastomoses, and bifurcations can make the access and adequate treatment of these areas challenging [5]. In addition, it is important to consider that bacteria can also be found inside the dentinal tubules, penetrating up to 1000 µm deep [6,7].

Lasers of the erbium family, specifically Er:YAG (2940 nm) and Er,Cr:YSGG (2780 nm), have been studied in the field of endodontics to perform laser-activated irrigation (LAI) as an alternative to passive ultrasonic irrigation (PUI) [8,9,10]. These lasers can create a micro-burst inside an irrigating solution due to their high absorption by water molecules. This phenomenon, known as cavitation, generates photomechanical shock waves that effectively remove the smear layer, resulting in clean, open dentinal tubules [11]. Consequently, there is a greater penetration of sodium hypochlorite (NaOCl) into the dentinal tubules and an improved biofilm removal [12].

The use of NaOCl in periapical surgical procedures may pose challenges due to its cytotoxic activity, as reported in the literature. When NaOCl diffuses into the periapical tissue, it can potentially cause a chemical burn and result in localized or extensive necrosis [13,14].

New strategies are needed to achieve effectiveness and safety in using NaOCl in periapical surgical procedures. In this regard, Iandolo et al. [15] proposed using NaOCl in gel form in the prepared cavity at the root end to prevent its extravasation into the surrounding tissues. The gel consistency of NaOCl provides a better control in the periapical preparation instead of the liquid consistency, avoiding extrusion to the periapical tissues. They demonstrated the improved penetration of the dentinal tubules by the gel when PUI was performed.

While PUI and traditional methods have been well studied for enhancing cleaning efficacy in apicectomy procedures, the application of LAI in the retrograde cavity remains underexplored. Given the laser’s potential to enhance the penetration and activation of irrigants in complex root canal systems, evaluating its effectiveness could provide valuable insights and potentially improve treatment outcomes in endodontic surgery.

This study aimed to assess the effectiveness of using Er,Cr:YSGG laser activation on NaOCl gel at concentrations of 0.5% and 2.5% to improve its penetration into the dentin walls. To determine its efficacy, the study also sought to compare this LAI technique with PUI using CLSM.

## 2. Materials and Methods

This study was approved by the Ethics Committee of the Universidad Católica de Valencia San Vicente Mártir with code number UCV/2022-2023/106. This study was carried out following the Preferred Reporting Items for Laboratory Studies in Endodontology (PRILE) 2021 guidelines [8].

### 2.1. Specimen Selection

Fifty freshly extracted mature single-root human teeth were selected, which were obtained due to periodontal problems. These teeth were stored in normal saline at room temperature. Before including the teeth in the study, all patients signed an informed consent form. To remove any remaining periodontal ligament debris and calculus from the root surface, the specimens were cleaned using endodontic tips and a Gracey 7/8 curette. The specimens were subjected to a disinfection process by being immersed in a 0.5% solution of chloramine-T for 48 h. After cleaning, the specimens were stored in a 10% formalin solution at 4 °C until use in the study.

The inclusion criteria involved selecting structurally sound single root canal and single root teeth, confirmed by radiographic examination. These teeth had to have a mature apex. On the other hand, the exclusion criteria were teeth with structural defects, root caries, very narrow and calcified root canals, open apex, root canals divided in the middle or apical third, external or internal root resorption, and teeth with vertical root fractures [16].

### 2.2. Chemo-Mechanical Preparation of the Root Canal

A standard chemo-mechanical preparation was performed [17], which was started by accessing the cavity using a no. 4 round bur. The cervical third was prepared with Gates–Glidden burs. Then, a size #10 Iso-K file was inserted into the canal until the tip of the file was visible through the apex. The working length of the canal was determined by subtracting 0.5 mm from this measurement. The root canals were instrumented using Protaper Gold files until a minimum apical size of ISO 25 was reached, corresponding to the F2 file. The irrigation protocol involved using 5.25% NaOCl between files, with a total volume of 5 mL, using a 30-gauge needle. After this, the root canals were irrigated with saline solution and 3 mL of 17% ethylenediaminetetraacetic acid (EDTA) for 60 s to remove the smear layer. A final irrigation step was performed using 2 mL of saline solution.

After chemo-mechanical preparation, the root canals were carefully dried with sterile F2 paper tips and sealed with resin cement Ah Plus and gutta-percha F2 using the single cone technique. In cases where the size and shape of the canal required it, lateral condensation was performed with an auxiliary gutta-percha cone. After the obturation procedure, the teeth were stored in a saline solution at room temperature for one week.

### 2.3. Retro-Cavity Preparation and NaOCl Gel Preparation

A 3 mm root end resection was performed [18] with a cylindrical multiblade bur, and then a retro-preparation was conducted to a depth of 3 mm using the Satelec P14D retrotype. A NaOCl solution with a gel-like consistency was prepared at two different concentrations—0.5% and 2.5%. In a previous study, the chemical composition of the gel used was described in detail [10]. To improve visibility under CLSM analysis, a 0.1% Rhodamine B dye was added to the NaOCl gel. This allowed for the better visualization and analysis of the penetration of the mixed NaOCl gel solution into the dentinal tubules.

### 2.4. Study Groups

**Figure 1:**
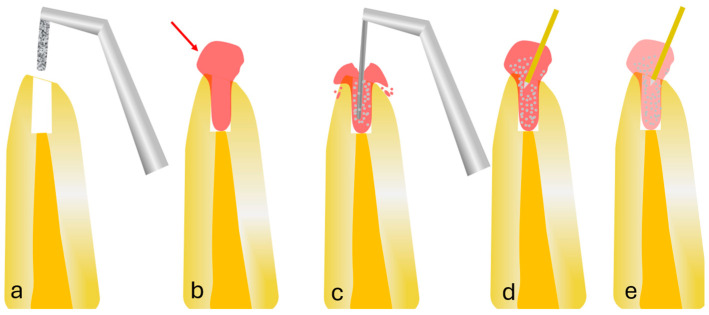
(**a**) Retro-cavity preparation after apicectomy, (**b**) NaOCl (2.5%) gel application without activation (red arrow represents the NaOCl gel with rhodamine dye), (**c**) PUI of 2.5% NaOCl gel, (**d**) Er,Cr:YSGG LAI of 2.5% NaOCl gel, (**e**) Er,Cr:YSGG LAI of 0.5% NaOCl gel. (Authored by Dr. Elafifi).

The teeth were then divided into four groups (Figure 1), as follows: Group 1—2.5% NaOCl gel without activation; Group 2—PUI group with activation using 2.5% NaOCl gel solution; Group 3—LAI group with activation using 2.5% NaOCl; and Group 4—LAI group with activation using 0.5% NaOCl gel solution. Five teeth were used as a control group and 15 were used in each treatment group. After retro-preparation, the retro-cavity was initially cleaned with 2 mL of sterile saline using a 30-gauge endodontic needle. All the study groups were conditioned with 17% EDTA and activated with a modified curved ultrasonic tip for 30 s and were then left for 30 s before being rinsed with 3 mL of sterile saline. Finally, the retro-cavity was dried using sterile paper cones. The protocols used for each group are described in detail below.

Group 1: The 2.5% NaOCl gel solution was applied to the retro-cavity and left to act for 60 s, before being rinsed with sterile saline and blotted dry with paper tips.

Group 2: The 2.5% NaOCl gel solution was activated using a modified ultrasonic tip for 30 s and was left to act for 30 s before being rinsed. An ultrasonic device was used for the PUI procedure. The device was equipped with a handpiece and was operated at a frequency of 30 kHz in endo mode, specifically at medium power.

Group 3: Three activation cycles were employed using the radial firing tip (RFT2) with a diameter of 200 µm, a length of 21 mm, and a calibration factor > 0.55. Each cycle consisted of a 5 s activation by dipping the tip into the 2.5% NaOCl gel and a 10 s pause during which the activated gel remained in place. The laser parameters were 0.75 W power, 50 Hz frequency, 0% air, and 0% water. The gel was replaced before beginning a new activation cycle. A pulsed Er,Cr:YSGG laser was used with a wavelength of 2780 nm for LAI.

Group 4: The same activation protocol as the third group was used, but the concentration of NaOCl gel (0.5%) was changed.

### 2.5. Confocal Laser Microscopy Analysis

**Figure 2:**
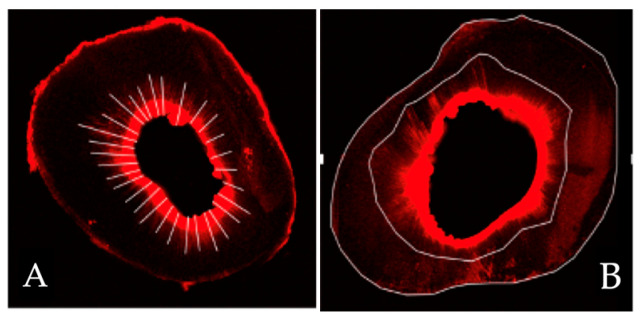
Representation of the measurement using Q-Path software. (**A**) various lines measuring the penetration depths all around the root canal to be able to determine the mean penetration depth; (**B**) a line defining all the penetration peaks and another line tracing the outer surface of the root to determine the penetration index.

**Figure 3:**
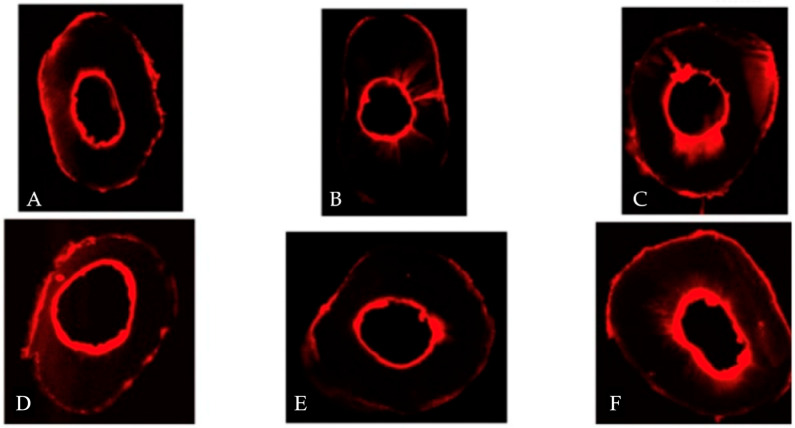
Sample confocal microscopy images of the different groups. The images represent the amount of penetration of the rhodamine dye from the root canal walls outwards towards the external root surface using different NaOCl gel activation protocols. (**A**–**D**) control group, (**B**–**E**) ultrasonic group, (**C**–**F**) laser group.

Samples were stored at 100% humidity and 36 °C for 7 days (as stated in a previous study [15]) prior to analysis. A 1-mm-thick slice was taken from the apex in cross-section using a cutting machine sprayed with water spray for cooling during the procedure. An independent, blinded evaluator studied the sections using confocal laser scanning microscopy at a wavelength of 545/740 nm. The penetration area was delineated using the Q-Path© software 0.4.3 and was automatically measured in µm^2^. The maximum penetration length was measured using the same software by drawing a line from the inner surface of the canal walls to the deepest point of irrigation via the outer surface of the root (Figure 2). The confocal microscopy images for different groups were obtained (Figure 3). Since not all the roots included in the study had the same surface area, we measured the four values mentioned above (buccal, lingual, mesial, and distal) and related them to the total surface area of the measured zone, which we call the penetration index.

### 2.6. Statistical Analysis

The data analysis was performed using the SPSS 23^©^ software with a 95% confidence level. If the *p* value was less than 0.05, the results were considered statistically significant compared to other groups. Kruskal–Wallis and Mann–Whitney tests were used to compare the results of the main variables, such as penetration index and mean penetration length.

## 3. Results

**Figure 4:**
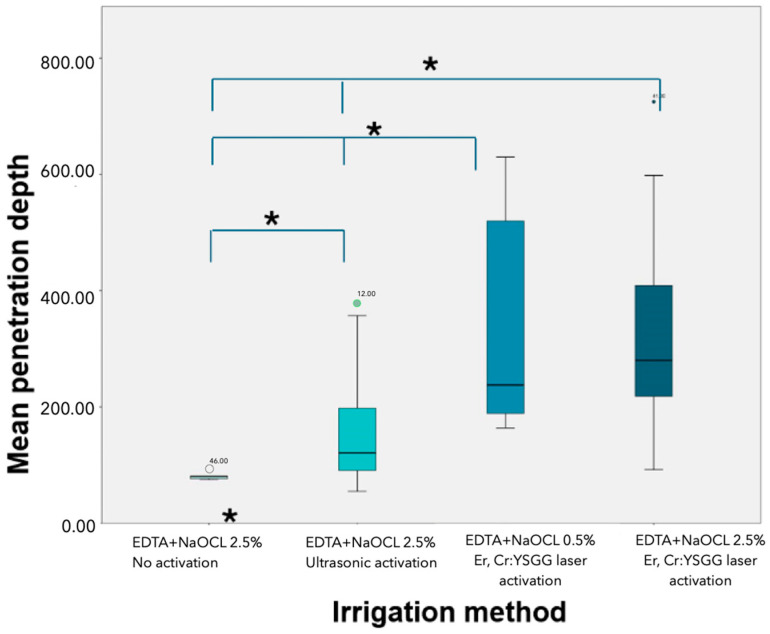
Box plot of the statistical difference between groups in terms of the mean penetration depth (the * indicates that the difference in the results between these groups is statistically significant).

**Figure 5:**
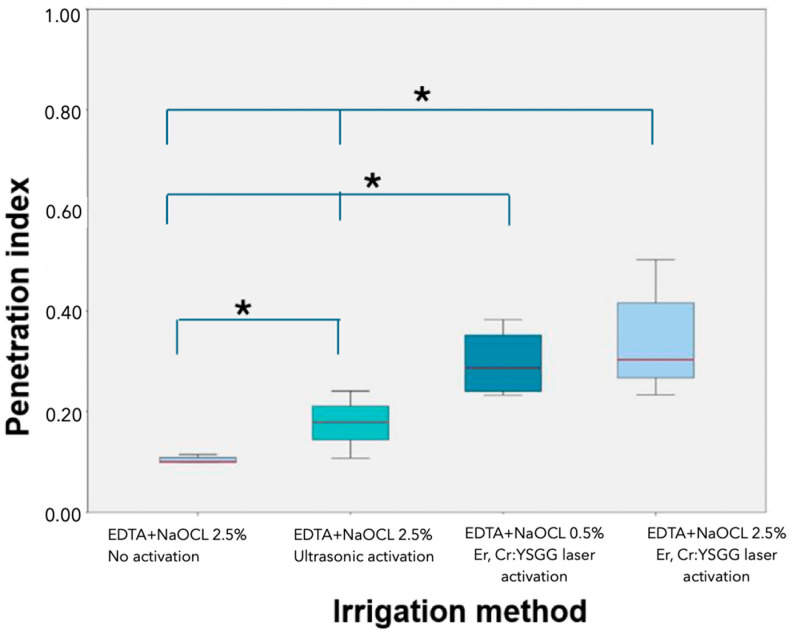
Box plot of the statistical difference between groups in terms of penetration index (the * indicates that the difference in the results between these groups is statistically significant).

The LAI groups showed significantly better results than the PUI group regarding hypochlorite gel penetration into the dentinal tubules (*p* < 0.05). No significant differences were observed between the two different concentrations of NaOCl (2.5% and 0.5%) in the LAI groups (*p* > 0.05) (Table 1 and Table 2) (Figure 4 and Figure 5).

## 4. Discussion

High-intensity lasers have demonstrated a superior antimicrobial effect on infected root canals, and these can be similarly employed to decontaminate the retro-cavity after an apicoectomy [19,20].

According to reports [21], whole-tissue lasers such as Er:YAG and Er,Cr:YSGG have proven effective in root-end re-sectioning. These lasers offer advantages such as improved sealing of the dentin surface, reduced crack formation, and the absence of smear layer formation. However, to our knowledge, no research has been conducted to evaluate how the Er,Cr:YSGG laser impacts NaOCl activation in the retro-cavity, specifically in relation to dentin penetration.

Our study’s findings agree with previous research by Iandolo et al. [15], which showed that NaOCl gel penetration was improved by PUI compared to the control group without activation. Furthermore, our study revealed an even higher penetration capability when the Er,Cr:YSGG laser was used, which supports previous studies indicating smear layer removal and a higher degree of dentinal tubule opening [22].

In addition, we observed a reduction in NaOCl extrusion from the retro-cavity during LAI in contrast to PUI. This difference can be attributed to LAI generating more effective cavitation than PUI [23,24]. Unlike the physical displacement of the liquid in the ultrasonic irrigation group, the laser group uses the absorption of high pulse energies by the water molecules, resulting in the formation of bubbles and their subsequent bursting [25]. This process leads to vaporization and the generation of a photoacoustic shock wave via the 200 µm RFT. Tip design plays a significant role in obtaining better sidewall disinfection during LAI. The radial energy emission from the tip allows for a more effective and complete lateral disinfection. This design ensures that the laser energy is evenly distributed in a 360-degree pattern, targeting all the root canal areas, and promoting comprehensive disinfection.

The novelty of this study lies in offering a safer way to use NaOCl in periapical microsurgery by using lower concentrations, which are laser-activated, to decrease its harmful effects on cells, while retaining its ability to fight microbes, which is comparable to higher concentrations of NaOCl. In the experimental groups, we used a 2.5% NaOCl solution, as suggested by Betancourt et al. [24], who found that this concentration effectively decreased the *E. faecalis* biofilm. In addition, we achieved results comparable to the study by Iandolo et al. [15], suggesting that the penetration capacity of NaOCl is not influenced by its concentration. Consequently, we chose not to use the 5.25% concentration to standardize the concentration and to eliminate any non-relevant variables, since our study is not microbiological.

Regarding the study variables, we modified our methodology compared to the previous study [15], which only measured a maximum penetration length in each aspect (buccal, lingual, mesial, and distal). Using this approach can provide us with more reliable results and an accurate estimate of the penetration capacity of the gel.

In our study, we introduced a revised method of LAI, which we developed based on the initial experiments we conducted. This modified protocol aims to maintain sufficient NaOCl and provide a 10 s interval for dissolution and penetration. By implementing activation cycles lasting 5 s, we ensure that the excessive evaporation of the rinsing agent is avoided, thereby guaranteeing the desired results. Indeed, the interrupted laser cycles implemented in our study had a total time of 45 s, which is shorter than the total duration of the ultrasound protocol, which usually lasts 60 s. By reducing the time required for laser cycles, we seek to optimize efficiency while achieving comparable results. This approach allows for a more time-effective treatment while maintaining the necessary efficiency in cleaning and disinfection. We chose this type of laser based on the flexible radial firing tip available for this device, which allows for high versatility and accessibility to the retro-cavity if applied in a clinical situation.

With the superior results obtained with the laser, it is interesting to encourage its use due to its additional antimicrobial effect through the direct activation of the disinfectant solution [26]. Moreover, the application of an all-tissue laser in periapical surgery simplifies the procedure by offering versatility in terms of incision, bone ablation, hemostasis, decontamination of periapical defects, and even performing root-end resections using the same laser [27]. However, to study the clinical significance and potential for an improvement in the success rates of endodontic apical microsurgery, randomized controlled clinical trials are needed.

This study has potential limitations such as the lack of microbiological analysis to be able to confirm the efficacy of this approach to reduce the bacterial count at the reported penetration depth; another limitation is that the sample teeth used were not from the same age group, thus the diameter and number of dentinal tubules can vary, which might affect the NaOCl penetration independently from the activation technique.

## 5. Conclusions

The use of the Er,Cr:YSGG laser to activate NaOCl gel in retro-cavity preparation leads to a higher penetration of NaOCl both in terms of area and length in the dentinal tubules compared to PUI. The concentration of the NaOCl gel did not affect its depth penetration. These findings support randomized controlled clinical trials with microbial analysis and the evaluation of the long-term clinical success.

## Figures and Tables

**Table 1:** *p* values in penetration index. * statistically significant difference at level 0.05; the penetration index value of each group is reported in µm^2^.

Irrigation Technique	Irrigation Technique (Comparison Groups)	*p*-Value
EDTA + NaOCl 2.5% without activation (0.10 ± 0.01 µm^2^)	EDTA +NaOCl 2.5% ultrasonic activation	0.001 *
	EDTA + NaOCl 0.50% laser activation	<0.001 *
	EDTA + NaOCl 2.5% laser activation	<0.001 *
EDTA + NaOCl 2.5% ultrasonic activation (0.18 ± 0.03 µm^2^)	EDTA + NaOCl 0.50% laser activation	<0.001 *
	EDTA + NaOCl 2.5% laser activation	<0.001 *
EDTA + NaOCl 0.50% laser activation (0.30 ± 0.03 µm^2^)	EDTA + NaOCl 2.5% laser activation (0.37 ± 0.08 µm^2^)	0.074

**Table 2:** *p* values in mean penetration length. * statistically significant difference at level 0.05; the mean penetration length of each group is reported in µm.

Irrigation Technique	Irrigation Technique (Comparison Groups)	*p*-Value
EDTA + NaOCl 2.5% without activation (81.11 ± 8.61 µm)	EDTA +NaOCl 2.5% ultrasonic activation	0.098
	EDTA + NaOCl 0.50% laser activation	<0.001 *
	EDTA + NaOCl 2.5% laser activation	<0.001 *
EDTA +NaOCl 2.5% ultrasonic activation (157.85 ± 54.81 µm)	EDTA + NaOCl 0.50% laser activation	0.001 *
	EDTA + NaOCl 2.5% laser activation	0.001 *
EDTA + NaOCl 0.50% laser activation (327.44 ± 102.80 µm)	EDTA + NaOCl 2.5% laser activation (327.15 ± 93.61 µm)	0.683

## Data Availability

The data that supports this study are available from the Catholic University of Valencia. Restrictions apply to the availability of these data, which were used under license for this study.

## References

[1] von Arx T., Jensen S.S., Janner S.F., Hänni S., Bornstein M.M. (2019). A 10-year Follow-up Study of 119 Teeth Treated with Apical Surgery and Root-end Filling with Mineral Trioxide Aggregate. J. Endod..

[2] Serrano-Gimenez M., Sanchez-Torres A., Gay-Escoda C. (2015). Prognostic factors on periapical surgery: A systematic review. Med. Oral Patol. Oral Cir. Bucal.

[3] Gomes B.P.F.A., Aveiro E., Kishen A. (2023). Irrigants and irrigation activation systems in Endodontics. Braz. Dent. J..

[4] Siqueira J.F., Silva W.O., Romeiro K., Gominho L.F., Alves F.R.F., Rôças I.N. (2024). Apical root canal microbiome associated with primary and posttreatment apical periodontitis: A systematic review. Int. Endod. J..

[5] Liu N., Li X., Liu N., Ye L., An J., Nie X., Liu L., Deng M. (2013). A micro-computed tomography study of the root canal morphology of the mandibular first premolar in a population from southwestern China. Clin. Oral Investig..

[6] George S., Kishen A., Song P. (2005). The Role of Environmental Changes on Monospecies Biofilm Formation on Root Canal Wall by *Enterococcus faecalis*. J. Endod..

[7] Wong D.T., Cheung G.S. (2014). Extension of Bactericidal Effect of Sodium Hypochlorite into Dentinal Tubules. J. Endod..

[8] Blanken J., De Moor R.J.G., Meire M., Verdaasdonk R. (2009). Laser induced explosive vapor and cavitation resulting in effective irrigation of the root canal. Part 1: A visualization study. Lasers Surg. Med..

[9] George R., Meyers I.A., Walsh L.J. (2008). Laser Activation of Endodontic Irrigants with Improved Conical Laser Fiber Tips for Removing Smear Layer in the Apical Third of the Root Canal. J. Endod..

[10] Betancourt P., Merlos A., Sierra J.M., Arnabat-Dominguez J., Viñas M. (2020). Er,Cr:YSGG Laser-Activated Irrigation and Passive Ultrasonic Irrigation: Comparison of Two Strategies for Root Canal Disinfection. Photobiomodulation Photomed. Laser Surg..

[11] De Groot S.D., Verhaagen B., Versluis M., Wu M., Wesselink P.R., Van Der Sluis L.W.M. (2009). Laser-activated irrigation within root canals: Cleaning efficacy and flow visualization. Int. Endod. J..

[12] Galler K.M., Grubmüller V., Schlichting R., Widbiller M., Eidt A., Schuller C., Wölflick M., Hiller K., Buchalla W. (2019). Penetration depth of irrigants into root dentine after sonic, ultrasonic and photoacoustic activation. Int. Endod. J..

[13] Mohammadi Z. (2008). Sodium hypochlorite in endodontics: An update review. Int. Dent. J..

[14] Guivarc’H M., Ordioni U., Ahmed H.M.A., Cohen S., Catherine J.-H., Bukiet F. (2017). Sodium Hypochlorite Accident: A Systematic Review. J. Endod..

[15] Iandolo A., Abdellatif D., Barbosa A.F.A., Scelza G., Gasparro R., Sammartino P., Silva E.J.N.L. (2022). Confocal laser scanning microscopy evaluation of roots subjected to activation protocol in endodontic microsurgery. Aust. Endod. J..

[16] Susila A., Minu J. (2019). Activated Irrigation vs. Conventional non-activated Irrigation in Endodontics—A Systematic Review. Eur. Endod. J..

[17] Alegre A.C., Verdú S.A., López J.I.Z., Alcina E.P., Climent J.R., Sabater A.P. (2022). Intratubular penetration capacity of HiFlow bioceramic sealer used with warm obturation techniques and single cone: A confocal laser scanning microscopic study. Heliyon.

[18] Degerness R., Bowles W. (2008). Anatomic Determination of the Mesiobuccal Root Resection Level in Maxillary Molars. J. Endod..

[19] Sohrabi K., Sooratgar A., Zolfagharnasab K., Fard M.J.K., Afkhami F. (2015). Antibacterial Activity of Diode Laser and Sodium Hypochlorite in Enterococcus Faecalis-Contaminated Root Canals. Iran. Endod. J..

[20] Vieira G.C., Antunes H.S., Pérez A.R., Gonçalves L.S., Antunes F.E., Siqueira J.F., Rôças I.N. (2018). Molecular Analysis of the Antibacterial Effects of Photodynamic Therapy in Endodontic Surgery: A Case Series. J. Endod..

[21] Berbert F.L.C.V., de Faria-Júnior N.B., Tanomaru-Filho M., Guerreiro-Tanomaru J.M., Bonetti-Filho I., Leonardo R.d.T., Marcantonio R.A.C. (2010). An in vitro evaluation of apicoectomies and retropreparations using different methods. Oral Surg. Oral Med. Oral Pathol. Oral Radiol. Endod..

[22] Montero-Miralles P., Torres-Lagares D., Segura-Egea J., Serrera-Figallo M., Gutierrez-Perez J., Castillo-Dali G. (2018). Comparative study of debris and smear layer removal with EDTA and Er,Cr:YSGG laser. J. Clin. Exp. Dent..

[23] Peeters H.H., Gutknecht N. (2014). Efficacy of laser-driven irrigation versus ultrasonic in removing an airlock from the apical third of a narrow root canal. Aust. Endod. J..

[24] Betancourt P., Sierra J.M., Camps-Font O., Arnabat-Domínguez J., Viñas M. (2019). Er,Cr:YSGG Laser-Activation Enhances Antimicrobial and Antibiofilm Action of Low Concentrations of Sodium Hypochlorite in Root Canals. Antibiotics.

[25] Badami V., Akarapu S., Kethineni H., Mittapalli S.P., Bala K.R., Fatima S.F., Akarapu D. (2023). Efficacy of Laser-Activated Irrigation Versus Ultrasonic-Activated Irrigation: A Systematic Review. Cureus.

[26] Cheng X., Guan S., Lu H., Zhao C., Chen X., Li N., Bai Q., Tian Y., Yu Q. (2012). Evaluation of the bactericidal effect of Nd:YAG, Er:YAG, Er,Cr:YSGG laser radiation, and antimicrobial photodynamic therapy (aPDT) in experimentally infected root canals. Lasers Surg. Med..

[27] van As G. (2004). Erbium lasers in dentistry. Dent. Clin. N. Am..

